# Improved haplotype-based detection of ongoing selective sweeps towards an application in Arabidopsis thaliana

**DOI:** 10.1186/1756-0500-4-232

**Published:** 2011-07-05

**Authors:** Torsten Günther, Karl J Schmid

**Affiliations:** 1 Institute of Plant Breeding, Seed Science and Population Genetics, University of Hohenheim, Stuttgart, Germany

## Abstract

**Background:**

The increasing amount of genome information allows us to address various questions regarding the molecular evolution and population genetics of different species. Such genome-wide data sets including thousands of individuals genotyped at hundreds of thousands of markers require time-efficient and powerful analysis methods. Demography and sampling introduce a bias into present population genetic tests of natural selection, which may confound results. Thus, a modification of test statistics is necessary to introduce time-efficient and unbiased analysis methods.

**Results:**

We present an improved haplotype-based test of selective sweeps in samples of unequally related individuals. For this purpose, we modified existing tests by weighting the contribution of each individual based on its uniqueness in the entire sample. In contrast to previous tests, this modified test is feasible even for large genome-wide data sets of multiple individuals. We utilize coalescent simulations to estimate the sensitivity of such haplotype-based test statistics to complex demographic scenarios, such as population structure, population growth and bottlenecks. The analysis of empirical data from humans reveals different results compared to previous tests. Additionally, we show that our statistic is applicable to empirical data from *Arabidopsis thaliana*. Overall, the modified test leads to a slight but significant increase of power to detect selective sweeps among all demographic scenarios.

**Conclusions:**

The concept of this modification might be applied to other statistics in population genetics to reduce the intrinsic bias of demography and sampling. Additionally, the combination of different test statistics may further improve the performance of tests for natural selection.

## Background

The recent advent of genome-wide surveys of genetic variation provides the opportunity to study genome-wide patterns of selection in model species. Such genome-wide scans detected new candidate regions for positive selection as well as previously identified target genes for selection, which included the lactase gene in European humans [1] or *FRIGIDA *in *Arabidopsis thaliana *[2].

Based on the assumption that the frequency of a new advantageous allele increases rapidly and that extended linkage disequilibrium (LD) around the selected site is expected [3,4], several tests for selective sweeps were designed in the last years [1,2,5-9]. The power of detecting selection with these haplotype-based tests was estimated to be higher than with frequency-based statistics as Tajima's *D *[1]. Although it is known that demographic history may cause a similar departure from the neutral model than selective sweeps and that test statistics are highly sensitive to these scenarios [10-14], only the pairwise haplotype sharing score (PHS, [2]) corrects for demographic history and relatedness. Unfortunately, because of pairwise comparisons between individuals for each allele, calculating the PHS has a complexity of *O*(*n*^2^) and is infeasible for large present and future data sets. However, since demography and unequally related individuals introduce a bias and potentially cause flawed results in sweep detection, a correction is required. Population structure also confounds genome-wide association studies and several approaches were developed to circumvent these problems [15]. The ideal sample for an association study as well as for scans for selective sweeps consists of equally related individuals with a star-like phylogeny. For samples from natural populations this assumption is unrealistic.

In order to correct for demographic effects in haplotype-based detection of ongoing selective sweeps, we modified the integrated haplotype score (iHS) statistic introduced by Voight et al. [1] by weighting the contribution of each individual according to its genetic similarity to all other individuals in the sample. Closely related individuals generally share more alleles and haplotypes because of common ancestry. The concept of weighting to account for an unequally related sample is already established in other fields of evolutionary analysis. It was introduced as branch-proportional sequence weighting in the construction of sequence profiles from homologous proteins [16] and also has been shown to improve the accuracy of multiple sequence alignments in CLUSTALW [17]. Here, we describe the weighted iHS (WiHS) method as an improved test statistic to detect ongoing selective sweeps. We utilize coalescent simulations of different complex demographic scenarios to estimate the detection power and the false discovery rate of the new method and compare it to existing methods. Finally, we apply the modified test statistic to empirical data from *Arabidopsis thaliana *and humans.

## Materials and methods

### Test statistic to detect selective sweeps

The new test statistic is based on the integrated haplotype score (iHS, [1]). The iHS is derived from the extended haplotype homozygosity (EHH, [4]) and assumes that selected haplotypes will be longer than the haplotypes around non-selected alleles in the same region because of hitchhiking of linked variation with the selected mutation. The EHH is defined as the probability that two haplotypes with the same core allele at position *x *are identical over the complete interval between the core site and a position *y*. The original EHH considers all individuals as equally weighted in the computation of the score.

We modified the EHH to account for unequally related individuals or population structure in the sample by utilizing a matrix of pairwise distances between all individuals. For the present paper, we calculated the squared genome-wide Hamming distance inferred from the genotypes, which performed well in accounting for relatedness in genome-wide association studies [18], but in general any distance metric is applicable. From pairwise distances we derive a measurement of the uniqueness, *U*, of each individual, *I*, to characterize the differences of an individual to a set of other individuals and then the contribution of each individual to the test statistic is weighted based on its uniqueness. We define *U *as

where *D_x_*(*I*) is the average pairwise distance of individual *I *to all other individuals carrying the same core allele at position *x *and *X *is the set of these individuals. Note that the sum of all uniquenesses for a certain allele is always , therefore only the relative weighting between individuals changes, which depends on the set of individuals carrying the same core allele at position *x*. Such weighting leads to a higher effect of less close related individuals on the test score and thus aims to reduce the bias in the sweep detection caused by unequal relatedness in the sample. The weighted EHH (wEHH) at position *y *is then computed for all sites with a minor allele frequency of more than 5% as

where *h *is a set of individuals carrying the same haplotype between *x *and *y*, *H *is the set of all haplotypes, *m *is the number of individuals carrying the same core allele at position *x *and *n *is the total sample size. For the classical EHH calculation, Σ*U*(*I_i_*) is replaced by the constant 1.

The subsequent steps are then identical to the original iHS approach [1]. We integrate under the wEHH decay around the specified core allele until wEHH reaches 0.05 using the trapezoidal rule. The integrated wEHH (iwEHH) is the sum of this integral in both directions from the core allele using distances on a genetic map to the core site to correct for different local recombination rates. The iwEHH is computed for both, the ancestral and derived allele, at position *x*, resulting in iwEHH_A _and iwEHH_D_, respectively. The unstandardized test statistic of the weighted integrated haplotype score, WiHS hereafter, is then computed as

This score is negative if the derived haplotype is larger than the ancestral haplotype and positive if the ancestral haplotype is larger. Since young, low frequency haplotypes are generally longer than old, high frequency haplotypes, we obtain a standardized score for the allele frequency *f *as follows

where mean*_f _*is the mean score of all sites with the frequency *f *and *SD_f _*is the associated standard deviation.

Python scripts used for the tests are available from http://evoplant.uni-hohenheim.de

### Simulation of selective sweeps

To assess the power of our method to detect selective sweeps, we applied it to simulated data sets. We simulated populations using the coalescent simulator *msms *[19] and sampled 100 chromosomes of 2 Mbp from the data. 4,000 SNPs with a minor allele frequency ≥ 0.05 were randomly selected from all simulated mutations. This sampling scheme corresponds roughly to the SNP density analyzed with SNP arrays in *A. thaliana *[20]. For each simulation run, a single site under positive selection without recurrent mutations was simulated and realistic mutation and recombination rates from *A. thaliana *were used [21,22]. The simulation parameters are summarized in Table 1. To compare the new method to other haplotype based tests for selective sweeps, we additionally computed the unweighted iHS [1] and the pairwise haplotype sharing score (PHS, [2]) for the simulated data sets, using the same standardization for allele frequency in all tests. For all simulations, a constant recombination rate without recombination hotspots was assumed. The selection coefficient was set to 2*N_e_s *= 200, other values are mentioned in the corresponding sections of the paper.

**Table 1:** Parameters for the msms simulations

Parameter		Value
Sequence length	*l*	2,000,000 bp
Sample size	*n*	100
Population scaled mutation rate (per site)	*θ*	6 · 10^-3^
Population scaled recombination rate (per site)	*ρ*	8 · 10^-4^
Effective population size	*N_e_*	1,000
Number of sampled SNPs		4,000

**Figure 1:**
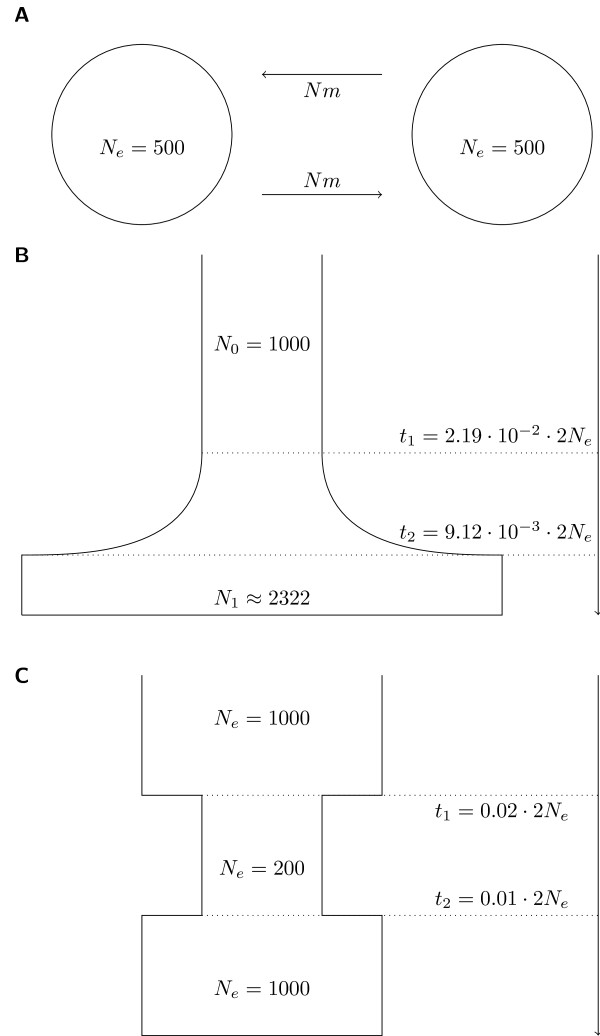
**Simulation models**. The demographic models used in the simulations included an island model (A), exponential population growth (B) and population bottleneck (C). The parameters of (B) are adjusted to a model by [23]. Time is given in units of 2*N_e _*generations.

To evaluate the performance of the modified test statistic on different demographic and selection scenarios, we simulated four different models: a panmictic population, an island model of two subpopulations with migration, an exponential population growth model which represents a realistic model for the European metapopulation of *A. thaliana *(growth model C from [23] with parameters scaled according to our population size), and a recent bottleneck (see Figure 1).

To assess whether high scoring SNPs cluster around the selected site, the absolute values of the scores were averaged in a window of ±25 SNPs around the selected site. These values were then used as final test statistic and compared to a null distribution estimated from neutral simulations of the panmictic model.

### Application to empirical data sets

We applied our new test statistic to two empirical data sets. The first data set was HapMap 2 [24] of the East Asian (JPT+CHB), European (CEU) and Yoruba (YRI) populations consisting of 120 chromosomes from each population. We included all SNPs for which an ancestral state was available from dbSNP 130 [25]. The estimated recombination rates were downloaded from the HapMap project and a polynomial curve was fitted to the markers for conversion between physical and genetic distances. Additionally, we analyzed SNP data from 199 *A. thaliana *accessions genotyped at approximately 220,000 SNP sites [20]. The alleles were polarized using the genome of the related species *Arabidopsis lyrata *[26]. For conversion from physical to genetic distances, we fitted a polynomial curve to 253 markers, for which physical and genetic positions are known [12]. All gene annotations were obtained from TAIR version 8 [27].

## Results

### Comparison of sweep statistics

**Figure 2:**
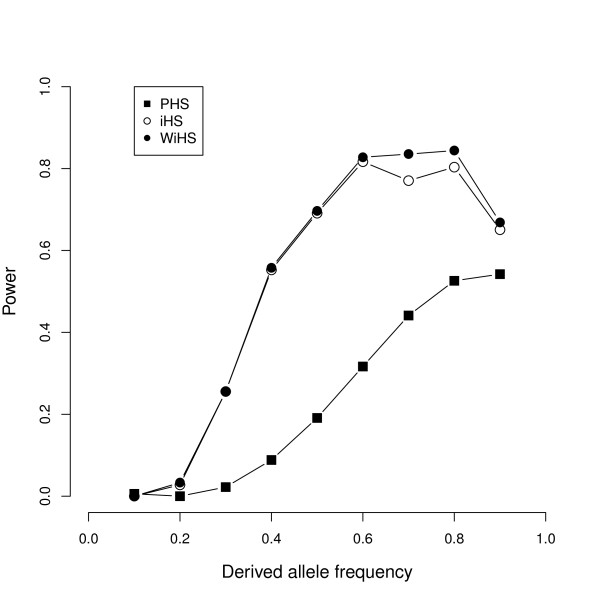
**Power to detect selective sweeps**. Detection power at a significance level of *α *= 001 based on the average score of all SNPs in a ±25 SNPs window around the selected site. For each allele frequency, 200 data sets were simulated and analyzed with all three tests. The null distribution was estimated in 1000 simulations without a selected site.

We restricted the comparison of our statistic to its closest relatives, the iHS and the PHS statistics. To our knowledge, the PHS is the only test with a correction for relatedness. As a basic model, we simulated a panmictic population. First, we checked the ranking of the selected sites based on their absolute scores. The mean rank of the causal SNP out of all 4000 SNPs was 195.9 (±17.6), 193.3 (±17.4) and 455.68 (±27.4) for iHS, WiHS and PHS, respectively. The difference between iHS and WiHS was not significant (pairwise Wilcoxon-test; *p *= 0.85). The relatively poor ranks show that a single SNP's score may be a bad identifier for a selective sweep. Therefore, we use the averaged absolute scores in a window of ±25 SNPs around the selected site as test statistic. This is similar to the approach chosen by Voight et al. [1]. It takes the hitchhiking variation into account, which is a important advantage if the causal site is not genotyped [1]. The power to detect a sweep using either of the three tests is highly variable across different allele frequencies (Figure 2). None of them is able to distinguish low frequency sweeps from neutral variation. The change in power is similar across different allele frequencies: both weighted and unweighted integrated scores show nearly identical graphs with a maximum power at an allele frequency between 60% and 80%, whereas the PHS test generally has a lower power for all frequencies and achieves its maximum between 80% and 90%. While the maximum power clearly differs at a significance level of *α *= 0.01 with WiHS having the highest and PHS having the lowest power, it is nearly identical for all three tests at *α *= 0.05 (data not shown), which is consistent with previous findings that the iHS has a high specificity [28].

A comparison of the iHS and WiHS tests shows that WiHS performs better than iHS for allele frequencies > 40% even in panmictic populations. As the power itself is based on a single stringent threshold for the test score based on a significance level, we compared the normalized test scores between iHS and WiHS directly and found that WiHS assigns higher absolute scores to the SNPs surrounding the selected site (pairwise Wilcoxon-test, *p *< 10^-15^). The scores around neutral sites are essentially identical for both tests (Additional File 1 Figure s1), which is expected for normalized scores. Thus, this difference demonstrates a better performance of WiHS in the detection of selective sweeps. While the absolute power decreased for selection weaker than 2*N_e_s *= 200 (Figure 4), a difference between iHS and WiHS was still observed and significant (pairwise Wilcoxon-test; *p *< 10^-6^, *p *< 10^-10 ^and *p *< 10^-15 ^for 2*N_e_s *= 50, 2*N_e_s *= 100 and 2*N_e_s *= 150, respectively). This difference is a consequence of the sampling process, because it is impossible to sample genetically equidistant individuals and therefore even random samples of a panmictic population exhibit a certain degree of structure. The weighting corrects for this bias and improves the power of selection tests.

**Figure 3:**
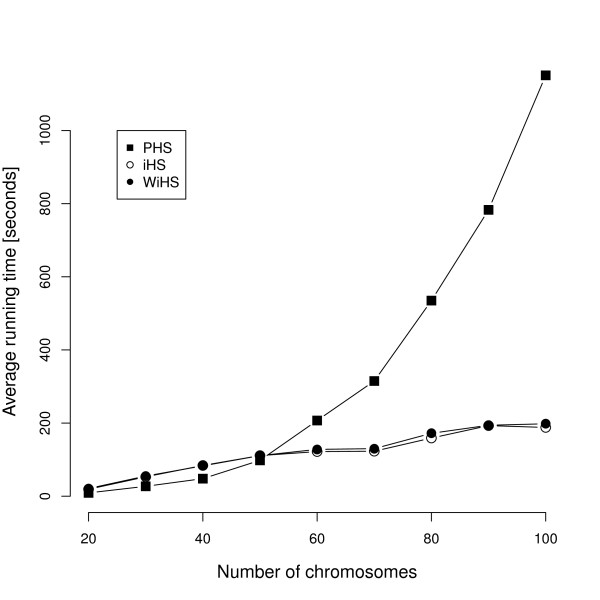
**Running time**. Average running times for the analysis of ten simulated data sets.

As the number of markers and individuals commonly used in sweep detection is rapidly growing, the running time of algorithms is becoming a limiting factor. We compared running times of all tests on simulated data sets and stepwise increased the number of analyzed chromosomes. Both integrated scores scale linearly, whereas PHS scales quadratically with the number of chromosomes (Figure 3). Since the PHS test is based on pairwise comparisons between individuals for each site, it is inefficient in running time and memory usage (not shown) for large data sets, while iHS and WiHS still have reasonable running times for data sets with thousands of individuals genotyped at hundreds of thousands of sites. However, sample sizes around 100 seem to be adequate for a reasonable power under panmictic scenarios, at least for the detection of strong selection (Additional File 1 Figure s2).

### Performance under different demographic scenarios

**Figure 4:**
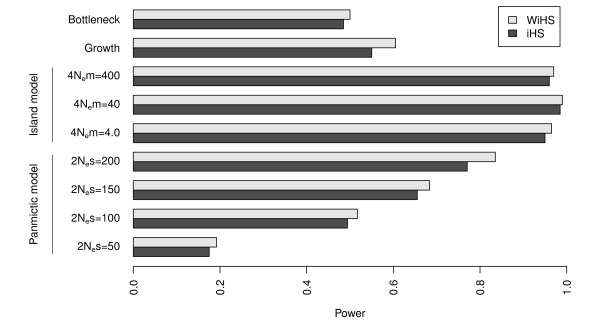
**Detection power under different demographic scenarios and parameters**. The power was estimated as the number of detected sweeps divided by the number of simulated sweeps. For each scenario, 200 simulations of sweeps with a present derived allele frequency between 60% and 80% were conducted.

The recent inclusion of selection in coalescent simulation software [19] permitted us to test WiHS under different demographic scenarios. First, an island model of two equally sized populations with varying migration rates was simulated (Figure 1A). Three different migration rates of 4*N_e_m *∈ {4, 40, 400} corresponding to a population differentiation of *F_ST _*between 0.0025 and 0.2 were simulated [29]. Higher levels of differentiation between populations are also possible, but in these cases a cross-population test (e.g. [8,9]) is more practical. The results suggest a marginally higher power of WiHS for all three migration rates (Figure 4) with significantly higher scores around the selected site (pairwise Wilcoxon-test; *p *< 10^-11^, *p *< 10^-15 ^and *p *< 10^-15 ^for 4*N_e_m *= 4.0, 4*N_e_s *= 40 and 4*N_e_m *= 400, respectively). The absolute power for all three migration rates is higher than observed under panmixia (Figure 4). Since there is no reason to expect such pattern, this may hint at an artifact in the simulations.

Additionally, a model of exponential population growth followed by a constant population size was simulated (Figure 1B). The model by [23] resembles the population history of European *A. thaliana *accessions. Therefore, we regard these simulations as a test case for the analysis of empirical data from *A. thaliana*. Compared to the panmictic model, the detection power was decreased by more than 20% (Figure 4). Nevertheless, WiHS had a power 5.5% higher than the power of iHS and the scores around the selected site were significantly higher for WiHS (pairwise Wilcoxon-test; *p *< 10^-9^). For the bottleneck model, a previously panmictic population was reduced to one fifth of its size with a later recovery to the original population size (Figure 1C). The bottleneck led to the strongest decrease in detection power (Figure 4), but WiHS still performed better and scored the SNPs in the sweep region higher (pairwise Wilcoxon-test; *p *< 10^-8^). For models with a non-constant population size, which is the case in the growth and bottleneck model, *msms *requires a defined start time of the selective sweep. The sweeps were initiated directly before the bottleneck or the start of population growth for the simulations above. Simulating different starts for the sweep showed no trend in the relation between time and detection power in both scenarios (Additional File 1 Figures s3, s4).

**Figure 5:**
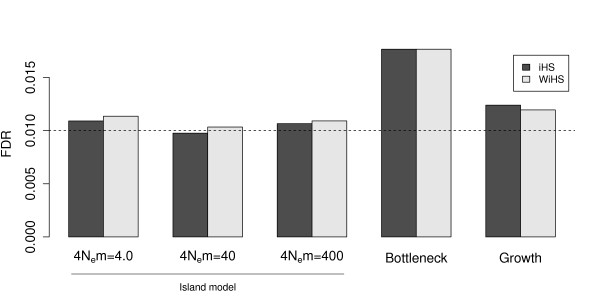
**False discovery rate under different demographic scenarios and parameters**. The FDR was estimated as the number of detected sweeps divided by the number of analyzed windows in simulations without selection. The dashed line represent the nominal significance level of 0.01. For each scenario, 200 simulations were conducted.

Biases introduced by demography are supposed to affect both the detection power and the number of false positives. To check for such biases, we used neutral simulations of all demographic models and calculated the false discovery rate (FDR) if the cutoffs were estimated from a panmictic model. The FDRs differ only marginally between iHS and WiHS (Figure 5). In general, the FDR at a nominal significance level of 0.01 is only slightly elevated, ranging form 0.010 to 0.018 for the island and bottleneck model, respectively (Figure 5).

### Selective sweeps in the HapMap data

To apply our new test statistic to empirical data, we re-analyzed 690,566, 748,881 and 709,542 HapMap2 SNPs [24] from the JPT+CHB, YRI and CEU populations, respectively. As numerous maps for positive selection in humans have been published earlier (reviewed by [30]), we were mainly interested in differences between the iHS and the WiHS tests instead of presenting an additional map of sweeps. We selected the 27 most relevant candidate genes or gene clusters that were previously identified as sweep regions and discussed in iHS studies of the particular populations [1,8,31]. Some of the candidate regions were regarded as sweep candidates in more than one population, therefore we investigated 34 regions in total (see Table 2). Scores were computed for all SNPs and then the average absolute score was estimated in a sliding window approach (50 SNPs window size, 20 SNPs offset between windows). Twenty out of the 34 regions were ranked among the genome-wide 5% highest scoring windows by both tests. We expected no complete overlap, since these candidates were identified in different data sets using different methods [30]. The WiHS test ranked 17 candidates better than the iHS while the latter test ranks eleven regions higher. The remaining six candidates were ranked identical. Summing up for all candidate regions, the ranking by WiHS was marginally improved in comparison to iHS (pairwise one-sided Wilcoxon-test, *p *< 0.1). The top 100 ranked windows differ only slightly between both tests (data not shown).

**Table 2:** Ranking of previously reported candidate genes in Human HapMap2 data

Gene(s)	Population	*p_iHS_*	*p_WiHS_*
*NCOA1, ADCY3*	YRI	0.003275	0.003189
*SNTG1*	YRI	0.023862	0.024517
*ITGB4BP, CEP2, SPAG4*	YRI	0.002392	0.002392
*SYT1*	YRI	0.115920	0.115265
*RSBN1*	YRI	0.062787	0.063043
*CPEB2*	YRI	0.252285	0.250491
*FZD6*	YRI	0.032490	0.032546
*CHST5, ADAT1, KARS*	YRI	0.146274	0.145904
*LARGE*	YRI	0.000598	0.000598
*NCDN, TEKT2*	CEU	0.001258	0.001228
*LCT*	CEU	0.000491	0.000552
*SNTG1*	CEU	0.001136	0.001136
*ITGB4BP, CEP2, SPAG4*	CEU	0.000583	0.000491
*CYP3A5*	CEU	0.597489	0.594758
*SLC24A5*	CEU	0.718933	0.714145
*OCA2*	CEU	0.091950	0.092533
*TYRP1*	CEU	0.008962	0.008900
*ERBB4*	CEU	0.117822	0.115827
*NRG3*	CEU	0.072522	0.072308
*ODF2*	CEU	0.874075	0.869595
*ACVR1*	CEU	0.067336	0.065095
*PDE11A*	CEU	0.026640	0.027438
*SNTG1*	JPT+CHB	0.006990	0.007055
*ITGB4BP, CEP2, SPAG4*	JPT+CHB	0.000755	0.000755
*CHST5, ADAT1, KARS*	JPT+CHB	0.095658	0.095790
*PDE11A*	JPT+CHB	0.081318	0.084665
*ERBB4*	JPT+CHB	0.020674	0.020641
*BLZF1, SLC19A2*	JPT+CHB	0.007613	0.007416
*SLC30A9*	JPT+CHB	0.012404	0.012929
*PCDH15*	JPT+CHB	0.001477	0.001477
*SLC44A5*	JPT+CHB	0.002658	0.002789
*SULT1C*	JPT+CHB	0.000525	0.000525
*ADH cluster*	JPT+CHB	0.023037	0.022938
*FLJ32745, EDAR*	JPT+CHB	0.765563	0.756571

The data was analyzed with both tests and the value represents the proportion of windows with a higher percentage of high scoring SNPs than a window overlapping with the candidate region.

### Selective sweeps in the A. thaliana data

**Figure 6:**
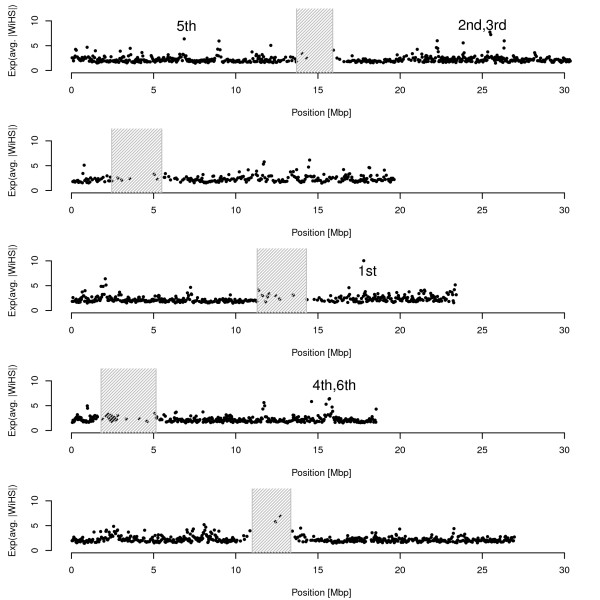
**WiHS results for the A. thaliana data**. WiHS was calculated for all SNPs and then the absolute scores were averaged in windows of 50 SNPs. To highlight the outstanding windows, the values were then exponentiated for this figure. The top six windows are labeled in the figure. The shaded regions denote the centromeric regions.

As the one of the highest power differences was observed for the growth model, the simulations indicate that WiHS offers an increased power for the analysis of data from *A. thaliana*. As a showcase for a genome-wide scan for selection in *A. thaliana*, we analyzed a genome-wide SNP data set of 220,000 SNPs from 199 accessions [20]. After WiHS was calculated for all SNPs, the genome was divided into non-overlapping windows of 50 SNPs and the absolute scores in these windows were averaged (Figure 6). A co-occurrence analysis of molecular function and biological process GO terms among the top 100 windows using GeneCoDis [32] revealed several over-represented categories (Additional File 1 Table s1). They include some categories that are of particular interest when looking for selection candidates. These categories comprise response to external and internal stimulus (e.g. auxin, light, salt stress) and flower development. The highest ranked term is the molecular function 'chitinase activity', some chitinases have been associated with pathogen response in *A. thaliana *[33].

In addition, a more detailed look was taken at the genes among the top 6 windows (Figure 6). The top ranked window overlaps with a region on chromosome 3 that was previously suggested as a sweep candidate [34]. This window includes *ARR5*, a gene involved in the cytokin signaling pathway, whose mutant shows a reduced rosette size and an increased sensitivity to red light. The windows ranked second and third contain *FKF1*, an F-box protein which is involved in the regulation of flower development and response to blue light, and *ANN5*, which is contributing in the response to heat, cold, salt stress, red light and water deprivation. Finally, the fourth and sixth ranked regions on chromosome 4 comprise *LUG1*, a regulator of *AGAMOUS *involved in the flower development.

## Discussion

### Detection of selective sweeps

Even in unstructured populations, sampling and relatedness introduce a bias into the sample. We improved the accuracy of detecting selective sweeps with haplotype based methods by weighting the contribution of each individual to the statistic according to its uniqueness in the sample. The improvement was observed in all simulated demographic scenarios including a panmictic population, a model of two subpopulations, exponential population growth and a population bottleneck. The increase of detection power of WiHS compared to iHS was less than expected but significant, reaching a maximum of 6.5%, 1%, 5.5% and 1.5% in the panmictic, island, population growth and bottleneck models, respectively. Simulation of different models and different model parameters, such as more severe bottlenecks, may give different results than the simulations in this study. The highest improvement was achieved in panmictic and growing populations. As the latter scenario was previously fitted to European accessions of *A. thaliana *[23], our improvement can result in additional sweep candidates for this species. While the detection power decreased in the more complex models, there was no significant increase of FDR if the sample was incorrectly assumed to arise from a panmictic population. As iHS and WiHS are genome-wide normalized scores, an excess of extreme scores and false positives under different demographic models is avoided.

The presented approach corrects for genome-wide IBD by upweighting more unique individuals in the sample. Since selective sweeps generate locally elevated IBD, which was suggested as a test for selection [35], one could also think of an opposite weighting based on local IBD. Local weighting would require the calculation of an IBD matrix for every single region, causing numerous pairwise comparisons between individuals and inflating the running time, which is beyond the scope of this paper.

Our simulation results extend the findings from previous studies for other test statistics [13,14,36-38] and show that haplotype-based tests are sensitive to demographic scenarios such as population structure and exponential growth. To identify candidates for selective sweeps, the search for outlier regions is commonly used, although they may represent the outliers of a neutral distribution [30]. Therefore, additional validation using tests based on other characters than haplotype length, such as site frequency spectrum [28,39-44] or population differentiation [44-46], will increase the reliability of sweep detections. Recently, compositions of different statistics have been shown to perform better in the detection of causal variants than each statistic separately [47-50] and the WiHS statistic might be included in such composite approaches as well and lead to a further improvement of these methods.

### Recent selection in empirical data sets

The analysis of empirical data sets provides an insight into the effect of the modification under real conditions. Among the top scoring windows of the HapMap data, some prominent candidate regions were found, such as *LCT *for lactose metabolism, *TYRP1 *for skin pigmentation and *SPAG4 *for sperm motility. Most but not all of these genes ranked better by WiHS, so we found only a weak significance. We are aware of the fact that some of these genes represent only candidates for positive selection that have not been validated. The trend suggests that general long-haplotype pattern in these regions is better detected by the WiHS and it is still possible that the ranking generated by WiHS is more accurate in the identification of selective sweeps.

The *A. thaliana *results revealed some promising candidates for selective sweeps. As the windows are still quite big, looking for particular candidate genes in these regions remains some kind of fishing in murky waters. Therefore, we leave the identification of sweep candidates to further studies, which employ a combination of different tests and use a more precise estimation of the genetic map. However, the simulations and the detection of some interesting genes in our preliminary scan suggest that WiHS is useful for the detection of selective sweeps in *A. thaliana*.

## Conclusions

Next-generation sequencing projects will provide sufficiently large data sets for the genome-wide detection of natural selection in many species (e.g. 1000genomes.org, 1001genomes.org, The *Drosophila *Genetic Resource Panel). The upcoming flood of data demands for time efficient and accurate analysis methods. Several methods operate with an equal contribution of individuals, which means that all individuals in the sample are assumed to be statistically independent. As it is very likely that not all pairs of individuals share the same most recent common ancestor, the assumption of independence should be violated in most biological samples. Thereby, unequally related individuals introduce a minor but significant bias into analyses, because the contribution of closely related individuals is overestimated while the contribution of others is underestimated. Such bias may be increased by demographic history and population structure. Genome-wide marker data allow to assess the relationship between individuals. This information can be used to cope with the dependency and to reduce the bias in estimates by differentially weighting the contribution of each individual. This concept could be extended to other unweighted statistics in population genetics. The consistent improvement across all simulated scenarios shows the general positive effect of differential weighting. Nevertheless, the slight increase of power leaves room for further improvement in the calculation of weights for each individual and the incorporation of these weights in test statistics, and for the detection of selective sweeps in general.

## Competing interests

The authors declare that they have no competing interests.

## Authors' contributions

TG conceived the initial approach. TG and KS designed the experiments. TG conducted the experiments. Both authors interpreted the results and wrote the manuscript. All authors read and approved the final manuscript.

## Supplementary Material

Additional file 1 **Supplementary Information**. The Supplementary Informations include additional figures and tables. Click here for file
