# Supplementary material for: Improved haplotype-based detection of ongoing selective sweeps towards an application in Arabidopsis thaliana
Source: BMC Res Notes. 2011 Jul 5;4:232. doi: 10.1186/1756-0500-4-232 (PMC3148560; doi:10.1186/1756-0500-4-232)
Supplement: Additional file 1 — Supplementary Information. The Supplementary Informations include additional figures and tables. [file 1756-0500-4-232-S1.PDF]

Improved haplotype-based detection of ongoing selective sweeps towards an application in *Arabidopsis thaliana*

Supplementary Information

Torsten Günther and Karl J. Schmid
Institute of Plant Breeding, Seed Science and Population Genetics,
University of Hohenheim, Stuttgart, Germany

Supplementary Figures

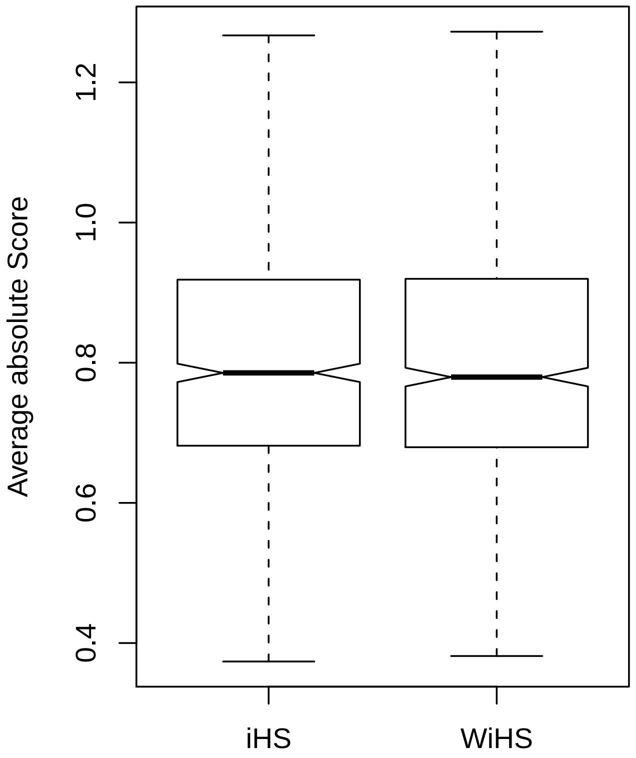

Figure 1: Average absolute score in a ±25 SNP window around neutral sites at an allele frequency between 65% and 75%.

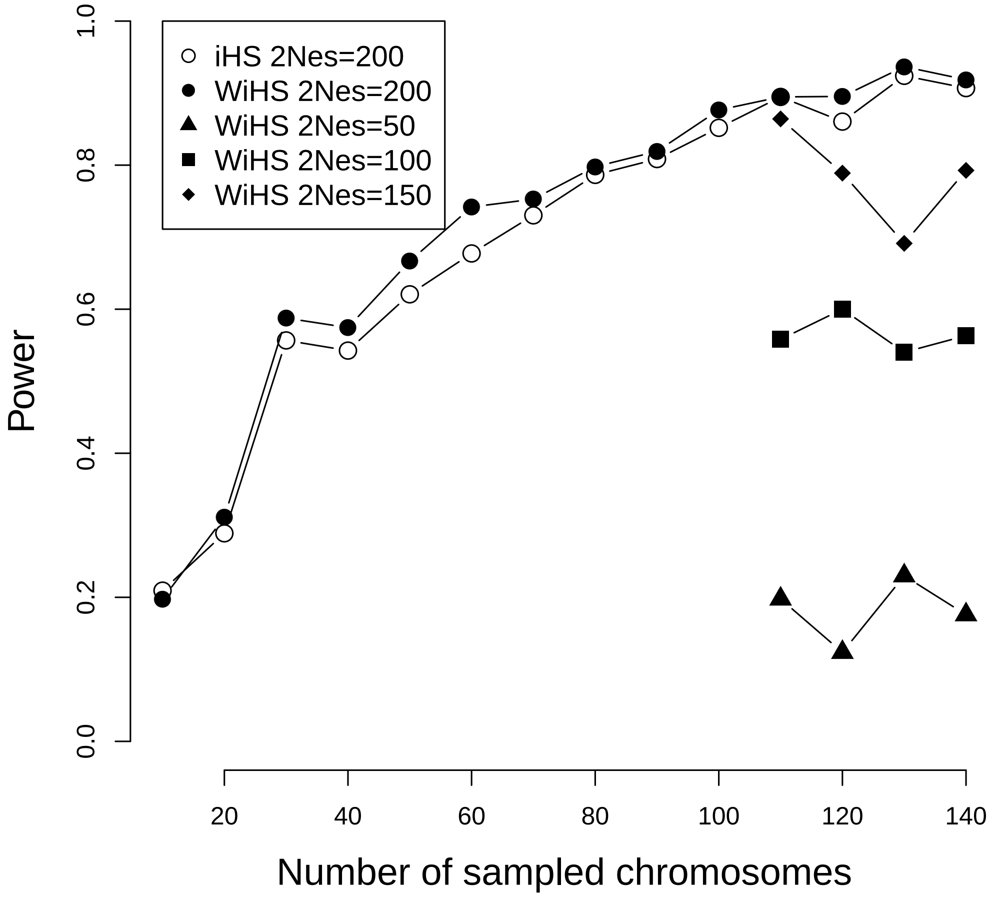

Figure 2: Detection power for different sample sizes at an allele frequency of 70% (panmictic model; 100 coalescent simulations per samplesize).

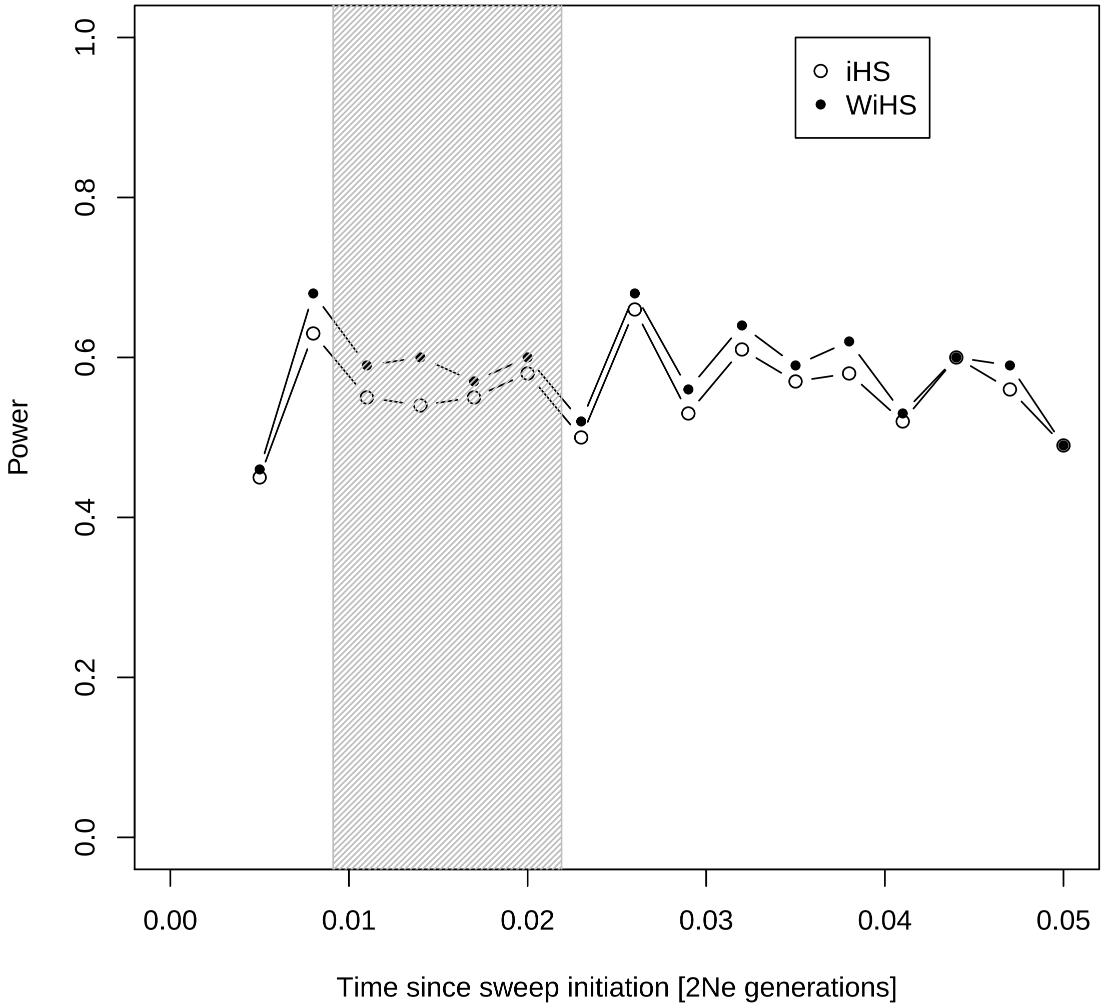

Figure 3: Detection power for different initiation times of the selective sweep in the growth model and for $2N_e s = 200$ (50 simulations per initiation time). The shaded area denotes the period of exponential population growth.

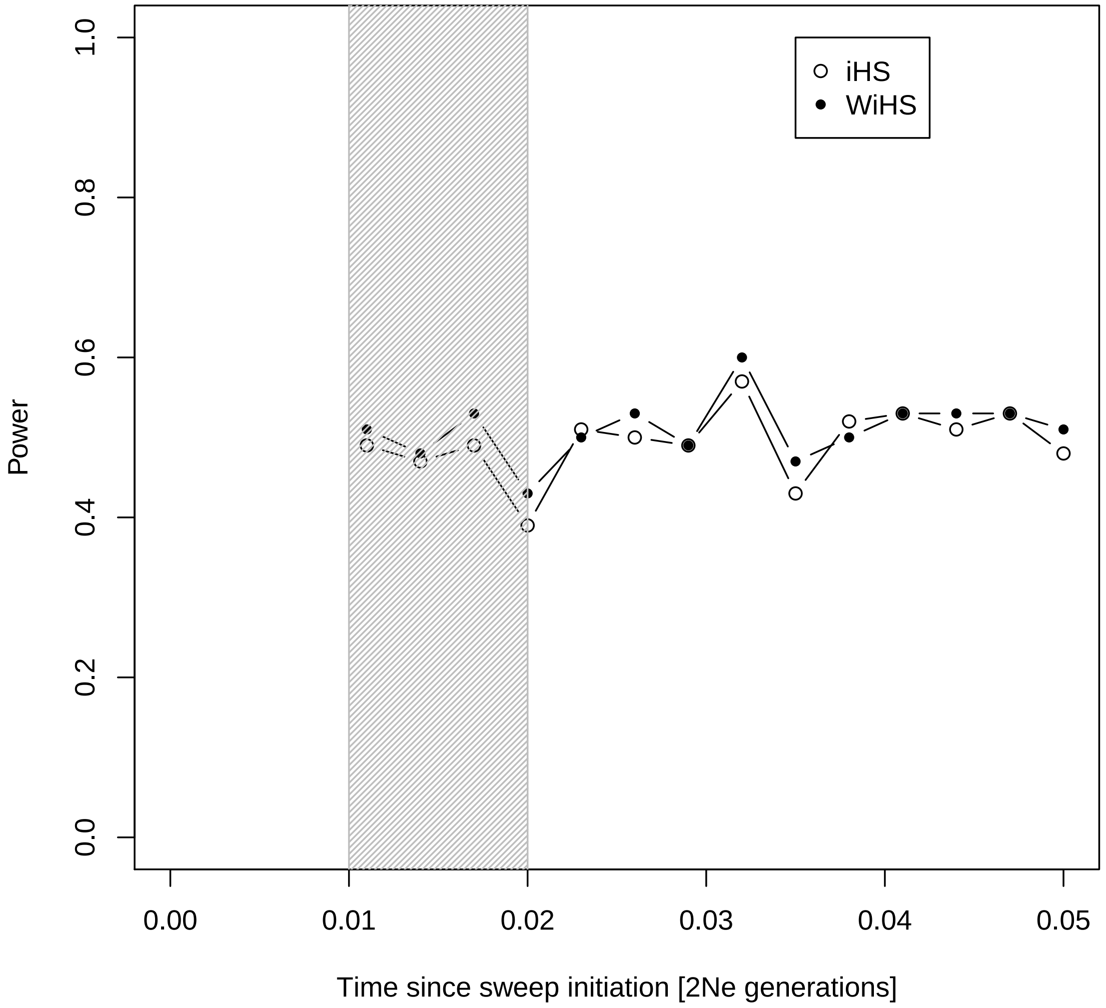

Figure 4: Detection power for different initiation times of the selective sweep in the bottleneck model and for $2N_e s = 200$ (50 simulations per initiation time). The shaded area denotes the period of the bottleneck.

Supplementary Table

Table 1: Co-occurence of GO categories in the top 100 WiHS windows of the *A. thaliana* dataset

Genes	NGR	NG	Chi*	Annotations
6 genes	12(23859)	6(934)	8.63566e-09	GO:0004568 :chitinase activity (MF)
2 genes	4(23859)	2(934)	0.0140184	GO:0010150 :leaf senescence (BP)
30 genes	1485(23859)	30(934)	0.0107164	GO:0005515 :protein binding (MF)
2 genes	5(23859)	2(934)	0.0278973	GO:0015380 :anion exchanger activity (MF)
1 genes	1(23859)	1(934)	0.00976029	GO:0009496 :plastoquinol-plastocyanin reductase activity (MF)
1 genes	1(23859)	1(934)	0.00976029	GO:0033962 :cytoplasmic mRNA processing body assembly (BP); GO:0031087 :deadenylation-independent decapping of nuclear-transcribed mRNA (BP); GO:0017148 :negative regulation of translation (BP)
1 genes	1(23859)	1(934)	0.00976029	GO:0042030 :ATPase inhibitor activity (MF)
1 genes	1(23859)	1(934)	0.00976029	GO:0016788 :hydrolase activity acting on ester bonds (MF); GO:0009628 :response to abiotic stimulus (BP); GO:0016036 :cellular response to phosphate starvation (BP); GO:0006796 :phosphate metabolic process (BP)
1 genes	1(23859)	1(934)	0.00976029	GO:0005515 :protein binding (MF); GO:0006350 :transcription (BP); GO:0004652 :polynucleotide adenylyltransferase activity (MF); GO:0043631 :RNA polyadenylation (BP); GO:0031123 :RNA 3'-end processing (BP)
1 genes	1(23859)	1(934)	0.00976029	GO:0004825 :methionine-tRNA ligase activity (MF); GO:0006431 :methionyl-tRNA aminoacylation (BP)
1 genes	1(23859)	1(934)	0.00976029	GO:0016787 :hydrolase activity (MF); GO:0015996 :chlorophyll catabolic process (BP); GO:0080124 :pheophytinase activity (MF)

1 genes	1(23859)	1(934)	0.00976029	GO:0003865 :3-oxo-5-alpha-steroid 4-dehydrogenase activity (MF); GO:0006665 :sphingolipid metabolic process (BP); GO:0010025 :wax biosynthetic process (BP); GO:0009922 :fatty acid elongase activity (MF); GO:0019166 :trans-2-enoyl-CoA reductase (NADPH) activity (MF)
5 genes	30(23859)	5(934)	0.0162641	GO:0016301 :kinase activity (MF); GO:0004712 :protein serine/threonine/tyrosine kinase activity (MF)
4 genes	403(23859)	4(934)	0.0412606	GO:0003824 :catalytic activity (MF)
1 genes	2(23859)	1(934)	0.0380725	GO:0009733 :response to auxin stimulus (BP); GO:0010114 :response to red light (BP); GO:0010218 :response to far red light (BP)
1 genes	2(23859)	1(934)	0.0380725	GO:0005488 :binding (MF); GO:0006810 :transport (BP); GO:0006839 :mitochondrial transport (BP); GO:0043132 :NAD transport (BP); GO:0051724 :NAD transporter activity (MF)
1 genes	2(23859)	1(934)	0.0380725	GO:0008270 :zinc ion binding (MF); GO:0016829 :lyase activity (MF)
1 genes	2(23859)	1(934)	0.0380725	GO:0008270 :zinc ion binding (MF); GO:0050897 :cobalt ion binding (MF); GO:0004591 :oxoglutarate dehydrogenase (succinyl-transferring) activity (MF)
1 genes	2(23859)	1(934)	0.0380725	GO:0006096 :glycolysis (BP); GO:0004618 :phosphoglycerate kinase activity (MF)
1 genes	2(23859)	1(934)	0.0380725	GO:0008805 :carbon-monoxide oxygenase activity (MF)
1 genes	2(23859)	1(934)	0.0380725	GO:0006629 :lipid metabolic process (BP); GO:0004806 :triglyceride lipase activity (MF); GO:0010027 :thylakoid membrane organization (BP); GO:0019433 :triglyceride catabolic process (BP); GO:0010150 :leaf senescence (BP)
1 genes	2(23859)	1(934)	0.0380725	GO:0006813 :potassium ion transport (BP); GO:0015079 :potassium ion transmembrane transporter activity (MF)

1 genes	2(23859)	1(934)	0.0380725	GO:0010090 :trichome morphogenesis (BP); GO:0009615 :response to virus (BP); GO:0051726 :regulation of cell cycle (BP); GO:0004693 :cyclin-dependent protein kinase activity (MF); GO:0009908 :flower development (BP); GO:0048366 :leaf development (BP); GO:0050792 :regulation of viral reproduction (BP)
1 genes	2(23859)	1(934)	0.0380725	GO:0003723 :RNA binding (MF); GO:0006396 :RNA processing (BP); GO:0004000 :adenosine deaminase activity (MF)
1 genes	2(23859)	1(934)	0.0380725	GO:0005509 :calcium ion binding (MF); GO:0006979 :response to oxidative stress (BP)
1 genes	2(23859)	1(934)	0.0380725	GO:0051302 :regulation of cell division (BP); GO:0035265 :organ growth (BP)
1 genes	2(23859)	1(934)	0.0380725	GO:0005515 :protein binding (MF); GO:0009733 :response to auxin stimulus (BP); GO:0005516 :calmodulin binding (MF)
1 genes	2(23859)	1(934)	0.0380725	GO:0016291 :acyl-CoA thioesterase activity (MF); GO:0006637 :acyl-CoA metabolic process (BP); GO:0030551 :cyclic nucleotide binding (MF)
1 genes	2(23859)	1(934)	0.0380725	GO:0016126 :sterol biosynthetic process (BP)
1 genes	2(23859)	1(934)	0.0380725	GO:0005515 :protein binding (MF); GO:0008270 :zinc ion binding (MF); GO:0003677 :DNA binding (MF); GO:0006355 :regulation of transcription DNA-dependent (BP); GO:0009911 :positive regulation of flower development (BP); GO:0009845 :seed germination (BP)
1 genes	2(23859)	1(934)	0.0380725	GO:0016462 :pyrophosphatase activity (MF)
1 genes	2(23859)	1(934)	0.0380725	GO:0005524 :ATP binding (MF); GO:0009790 :embryonic development (BP); GO:0009926 :auxin polar transport (BP); GO:0048364 :root development (BP); GO:0019827 :stem cell maintenance (BP); GO:0001708 :cell fate specification (BP); GO:0010305 :leaf vascular tissue pattern formation (BP)

1 genes	2(23859)	1(934)	0.0380725	GO:0005507 :copper ion binding (MF); GO:0046933 :hydrogen ion transporting ATP synthase activity rotational mechanism (MF); GO:0008270 :zinc ion binding (MF); GO:0006979 :response to oxidative stress (BP); GO:0050897 :cobalt ion binding (MF)
1 genes	2(23859)	1(934)	0.0380725	GO:0046686 :response to cadmium ion (BP); GO:0045449 :regulation of transcription (BP); GO:0003677 :DNA binding (MF); GO:0003700 :transcription factor activity (MF); GO:0009651 :response to salt stress (BP); GO:0009737 :response to abscisic acid stimulus (BP); GO:0009751 :response to salicylic acid stimulus (BP); GO:0009753 :response to jasmonic acid stimulus (BP); GO:0009733 :response to auxin stimulus (BP); GO:0009723 :response to ethylene stimulus (BP); GO:0009739 :response to gibberellin stimulus (BP)
1 genes	2(23859)	1(934)	0.0380725	GO:0016757 :transferase activity transferring glycosyl groups (MF); GO:0016758 :transferase activity transferring hexosyl groups (MF); GO:0010584 :pollen exine formation (BP)
1 genes	2(23859)	1(934)	0.0380725	GO:0005215 :transporter activity (MF); GO:0006857 :oligopeptide transport (BP); GO:0080055 :low affinity nitrate transport (BP); GO:0015706 :nitrate transport (BP); GO:0080054 :low affinity nitrate transmembrane transporter activity (MF)
2 genes	273(23859)	2(934)	0.0402171	GO:0009055 :electron carrier activity (MF)
1 genes	220(23859)	1(934)	0.0471382	GO:0004497 :monooxygenase activity (MF)

NGR = Number of annotated genes in the reference list (Total number of genes in the reference list)
NG = Number of annotated genes in the input list (Total number of genes in the input list)
Chi* = FDR corrected chi square pValue
